# Chicago sky blue 6B inhibits α-synuclein aggregation and propagation

**DOI:** 10.1186/s13041-022-00913-y

**Published:** 2022-03-28

**Authors:** Joo-Ok Min, Timo Strohäker, Byung-Chul Jeong, Markus Zweckstetter, Seung-Jae Lee

**Affiliations:** 1 Department of Biomedical Sciences, Neuroscience Research Institute, Seoul National University College of Medicine, 103 Daehak-ro, Jongro-gu, Seoul, 03080 Republic of Korea; 2 German Center for Neurodegenerative Diseases (DZNE), Von-Siebold-Str. 3a, 37075 Göttingen, Germany; 3 Department for NMR-Based Structural Biology, Max Planck Institute for Biophysical Chemistry, Am Faßberg 11, 37077 Göttingen, Germany; 4 Present Address: Nutritional Sciences and Toxicology Department, University of California Berkeley, Berkeley, USA

**Keywords:** Chicago sky blue 6B, α-synuclein, Protein aggregation, Aggregate propagation, Parkinson’s disease

## Abstract

Abnormal deposition of α-synuclein aggregates in Lewy bodies and Lewy neurites is the hallmark lesion in Parkinson’s disease (PD). These aggregates, thought to be the culprit of disease pathogenesis, spread throughout the brain as the disease progresses. Agents that inhibit α-synuclein aggregation and/or spread of aggregates would thus be candidate disease-modifying drugs. Here, we found that Chicago sky blue 6B (CSB) may be such a drug, showing that it inhibits α-synuclein aggregation and cell-to-cell propagation in both in vitro and in vivo models of synucleinopathy. CSB inhibited the fibrillation of α-synuclein in a concentration-dependent manner through direct binding to the N-terminus of α-synuclein. Furthermore, both seeded polymerization and cell-to-cell propagation of α-synuclein were inhibited by CSB treatment. Notably, CSB alleviated behavioral deficits and neuropathological features, such as phospho-α-synuclein and astrogliosis, in A53T α-synuclein transgenic mice. These results indicate that CSB directly binds α-synuclein and inhibits its aggregation, thereby blocking α-synuclein cell-to-cell propagation.

**Supplementary Information:**

The online version contains supplementary material available at 10.1186/s13041-022-00913-y.

## Introduction

Synucleinopathies are an ensemble of neurological diseases characterized by deposition of α-synuclein aggregates, in neurons in the case of Parkinson’s disease (PD) and dementia with Lewy bodies, and in oligodendrocytes in the case of multiple system atrophy [1–3]. α-Synuclein is a neuronal protein encoded by the *SNCA* gene that is localized mainly in presynaptic terminals [4]. Missense and multiplication mutations in *SNCA*, most of which promote aggregation of the protein, have been linked to familial forms of PD [5–7]. This, taken together with the fact that α-synuclein aggregates are the pathological hallmarks of PD, suggests that α-synuclein aggregation is a crucial event in the pathogenesis of PD.

PD is a progressive disease associated with multiple motor and non-motor symptoms [8, 9]. As the disease progresses, the symptoms become more severe and more complex. Pathologically, disease progression is associated with spread of α-synuclein aggregates. Initially, α-synuclein aggregates appear only at a few discrete regions in the brain, notably including the dorsal motor nucleus of the vagus and olfactory bulb [10], but subsequently spread to the midbrain and neocortical regions [10, 11]. This pattern of α-synuclein aggregate spread in the brain has been replicated in both mouse and nonhuman primate animal models, in which intracerebral injection of in vitro-generated fibrils or PD-derived Lewy body extracts results in brain-wide α-synuclein aggregation [12, 13]. Studies have suggested that direct cell-to-cell propagation of α-synuclein aggregates is responsible for this spread throughout the brain [14, 15].

α-Synuclein can spontaneously form prototypical amyloid fibrils through a nucleation-dependent process characterized by a lag phase followed by growth and equilibrium phases [16]. Addition of preformed fibrils eliminates the lag phase, a phenomenon referred to as templated seeding [17]. Templated seeding is considered the underlying principle for cell-to-cell aggregate propagation. Agents that inhibit both de novo and seeded aggregation would thus be considered candidate disease-modifying drugs.

In a separate study, we screened a small-molecule library for compounds that inhibit cell-to-cell propagation of α-synuclein. Among the “hits” identified by this search was Chicago sky blue 6B (CSB). Here, we show that CSB inhibits both de novo and seeded aggregation of α-synuclein in a cell-free system with purified proteins as well as in cell models. We found that this inhibition of aggregation is mediated by direct interactions between CSB and the N-terminus of α-synuclein. Notably, we showed that administration of CSB in A53T α-synuclein transgenic mice reduces α-synuclein deposition and gliosis and alleviates motor behavioral deficits.

## Materials and methods

### Materials

Rabbit anti-phospho-synuclein (S129) antibody (1:1000) and rabbit anti-glial fibrillary acidic protein (GFAP) polyclonal antibody (1:500), were obtained from Abcam (ab51253, Abcam) and Abcam (ab7260, Abcam), respectively. CSB was purchased from Tocris (0846, Tocris). Bafilomycin A1 was purchased from Calbiochem (196000, Calbiochem).

### Animals

Adult (6 months old) male and female PrP-A53T mice [G2-3 line, Tg(Prnp-SNCA*A53T)23Mkle/J, C57BL6/J strain] and their wild-type littermates (male and female) were used. All experimental procedures described below were performed in accordance with Seoul National University (Republic of Korea) Institutional guidelines for the care and use of experimental animals (IACUC number: SNU-190721-1-10).

### Cell culture

Cell lines stably expressing each half of the split Venus fluorescent system (V1S and SV2) for testing cell-to-cell transmission of α-synuclein were generated as described previously [18]. V1S and SV2 cells were subcultured in high-glucose Dulbecco’s modified Eagle medium (DMEM; SH30243.01, Hyclone) supplemented with 10% fetal bovine serum (FBS; SH30919.03, Hyclone) and 100 units ml^−1^ penicillin/streptomycin (GIB-15070-063, Gibco), additionally containing 200 μg ml^−1^ G418 (11811-031, Invitrogen). Cultures were maintained at 37 °C in a humidified 5% CO_2_ atmosphere, and the culture medium was replaced every 2 days.

Human neuroblastoma SH-SY5Y cells (CRL-2266, ATCC) were cultured and maintained at 37 °C in a humidified 5% CO_2_ atmosphere with media exchange every 2 days. Cells were differentiated by culturing in growth medium (DMEM + 10% FBS + 100 units ml^−1^ penicillin/streptomycin) containing 50 μM retinoic acid (R2625, Sigma Aldrich).

### High-content screening of drugs in an α-synuclein BiFC cell model

The effects of drugs on α-synuclein propagation were determined using a dual cell bimolecular fluorescence complementation (BiFC) method employing α-synuclein–conjugated split Venus fragments, which are expressed in separate cell lines (V1S and SV2 cells) and after cell-to-cell transfer, reassemble to form a fluorescent protein upon interactions of the conjugated protein [18]. V1S and SV2 cells were first cultured for 6 passages and then seeded onto a flat-bottom, 96-well black plate (655090, Greiner Bio-One) at 8 × 10^4^ cells per well. After 24 h, cells were incubated with 10 μM CSB at 37 °C for 48 h. CSB-treated cells were then incubated at 37 °C for 10 min with 10 μg/ml Hoechst 33342 (H1399, Invitrogen) to stain nuclei and with Topro-3 iodide (T3605, Invitrogen), diluted 1:1000, to detect dead cells. Topro-3 is only permeable in cells undergoing necrosis or apoptosis. Cells were washed once with DMEM, after which new medium without phenol red was added. Venus BiFC fluorescence in live cells was measured by imaging with an automated high-content screening reader (In Cell Analyzer 2200; GE Healthcare), and images were analyzed using the In Cell Developer Toolbox software. The concentration dependence of CSB-mediated modulation of α-synuclein cell-to-cell transmission was analyzed by incubating 1000 cells with different concentrations (1 nM, 10 nM, 0.1 μM, 1 μM and 10 μM) of CSB at 37 °C in a humidified CO_2_ environment. Three independent experiments were performed.

### Cell viability test

Cell viability was assessed by seeding differentiated SH-SY5Y cells overexpressing α-synuclein on 6-well plates and then adding CSB (1 nM, 10 nM, 0.1 μM, 1 μM, 10 μM) to each well and incubating at 37 °C for 2 days. After collecting cells by trypsinization, 2 μl of propidium iodide, which binds to DNA and is commonly used to detect dead cells, was added to 18 μl of cell suspension and the fluorescence of the resulting samples was measured using a LUNA cell counter (Logos Biosystems).

### Preparation of recombinant α-synuclein fibrils

Human α-synuclein was expressed in *Escherichia coli* BL21 DE3, and α-synuclein fibrils were prepared as described [19]. Briefly, human α-synuclein protein was induced by adding IPTG (isopropyl β-d-1-thiogalactopyranoside) to a final concentration of 0.1 mM and incubating cells for 3 h at 37 °C. The cells were centrifuged and resuspended in 20 mM sodium phosphate buffer (pH 7.4), then sonicated, boiled, and centrifuged at 10,000×*g* for 10 min at 4 °C. The supernatant was subjected to anion-exchange chromatography and Superdex-200 gel filtration column chromatography, after which fractions containing purified α-synuclein were immediately dialyzed and lyophilized. α-Synuclein polymerization reactions were performed using 200 μM α-synuclein monomers after filtration of the monomers by using a 100,000 MWCO (molecular weight cut-off) filters. α-synuclein was incubated with CSB or a vehicle control solution at 37 °C for 9 days with constant shaking at 1050 rpm in a Thermomixer C (5382000015, Eppendorf).

### Thioflavin-T binding assay

Amyloid fibril formation was assayed using thioflavin T (ThT), which binds to β-sheet–rich structures in the presence of amyloid fibrils. Specifically, 40 μl of 10 μM recombinant α-synuclein was added to 50 μl of a 10 μM Thio-T solution in glycine–NaOH (pH 8.5). After incubating for 5 min at room temperature, fluorescence was measured at 450 nm (excitation)/490 nm (emission) using a microplate reader (Synergy Neo, BioTek).

### Circular dichroism

For evaluation of the secondary structure of α-synuclein protein incubated with CSB, samples were diluted in PBS to 0.5 mg/ml and analyzed using a circular dichroism detector (Chirascan Plus).

### Transmission electron microscopy (TEM)

Samples of α-synuclein with CSB (0.1, 1, or 10 μM) or vehicle (DMSO) were applied to 200 mesh carbon-coated copper grids, then negative-stained by placing 10 μl of a 2% uranyl acetate solution on the grid for 5 min. Samples on grids were visualized using a JEM-1400 transmission electron microscope (JEOL).

### NMR spectroscopy

Expression and purification of ^15^N-labeled α-synuclein protein was performed as described previously [20]. For NMR experiments, ^15^N-labeled α-synuclein was dialyzed against buffer containing 100 mM NaCl, 50 mM HEPES, and 0.02% NaN_3_ (pH 7.4). ^1^H-^15^N heteronuclear single-quantum coherence (HSQC) experiments were recorded at 15 °C on a 600 MHz Bruker NMR spectrometer using the Bruker pulse program, hsqcetf3gpsi. ^1^H-^15^N HSQC experiments were performed using α-synuclein (80 μM) alone and in the presence of CSB at α-synuclein:CSB ratios of 1:0.1, 1:0.2, 1:0.5, 1:0.8, 1:1, 1:2 and 1:5. The combined ^1^H/^15^N chemical shift perturbation was calculated according to [(δ_H_)^2^ + (δ_N_/5)^2^]/2^1/2^, where δ_H_ and δ_N_ are the chemical shift values of ^1^H and ^15^N respectively. In addition, NMR signal intensities were fitted assuming a simple two-state exchange model, and the dissociation constant (K_d_) was derived from a global fit to the intensity decay curves of selected residues (residues 1–9, 12, 14–20) according to the following relationship:

$$\left( {1 - \frac{I}{{I_{0} }}} \right) = I_{\max } \left[ {\frac{{\left( {P_{0} + x + K_{d} } \right) - \sqrt {\left( {P_{0} + x + K_{d} } \right)^{2} - 4P_{0} x} }}{{2P_{0} }}} \right],$$

where *I* is the intensity value along the titration, *I*_*0*_ is the intensity value of the free state, *P*_*0*_ is the total amount of protein, *K*_*d*_ is the dissociation constant, and *x* is the concentration of α-synuclein (in μM) along the titration. Errors were estimated by evaluating the standard deviation of the intensity according to the following:

$$\sigma_{I} = \left( {\frac{I}{{I_{0} }}} \right)\sqrt {\left( {\frac{\sigma I}{I}} \right)^{{2{ }}} + { }\left( {\frac{{\sigma I_{0} }}{{I_{0} }}} \right)^{2} } ,$$

where *σI* and *σI*_0_ are the standard deviations of the noise in the spectra.

### Animal treatment

Transgenic mice (Tg) expressing A53T human α-synuclein under control of the cellular prion protein (PrP) promoter were used. Briefly, adult (6-month old) Tg mice and wild-type littermate controls were injected intraperitoneally with either CSB (20 mg/kg/day) or PBS once a week for 3 months.

### Immunohistochemistry

Adult mice were deeply anaesthetized with ketamine:Rompun (3.5:1; 2.5 μl g^−1^) and perfused with PBS followed by 4% paraformaldehyde (PFA; P6418, Sigma-Aldrich). Excised brains were post-fixed overnight in 4% PFA at 4 °C. Coronal section (40 μm) were cut with a vibratome, rinsed three times in PBS and then once with 3% H_2_O_2_ (Sigma-Aldrich) to quench endogenous peroxidase. Sections were washed with PBST (0.1% Triton X-100 in PBS) three times and incubated with blocking solution (4% bovine serum albumin in PBST) for 1 h at room temperature. Sections were incubated overnight in a mixture of rabbit anti-phospho-synuclein (S129) (ab51253, Abcam) and rabbit GFAP (ab7260, Abcam) primary antibodies, diluted 1:1,000 and 1:500, respectively, in blocking solution. The next day, sections were incubated with species-appropriate horseradish peroxidase (HRP)-conjugated secondary antibody for 1 h and 30 min at room temperature and then washed three times. Following incubation with an avidin–biotin complex (Vectastain ABC kit; PK6200, Vector Laboratories), immunocomplexes were visualized using 3,3′-diaminobenzidine (DAB; D5637, Sigma-Aldrich) with H_2_O_2_. Sections were mounted on gelatin-coated slides using Canada balsam (C1795, Sigma-Aldrich).

### Open field test

Locomotor activity in an open field (40 × 40 cm) was monitored in 9-month old mice using a video tracking system (EthoVision XT14; Noldus, Netherlands) that detects the center of gravity of the mouse in the arena. Spontaneous horizontal activity (total distance travelled) in the center and peripheral zone were measured for 10 min.

### Forelimb grip strength test

The forelimb grip strength test provides a measure of the neuromuscular activity of mice. In this test, the maximal force of the forelimbs of mice hanging on a metal grid surface was measured twice. Mean forelimb grip strength values were normalized to mouse body weight.

### Balance beam test

Motor balance and coordination were assessed by monitoring walking of familial Parkinson’s disease model mice across a beam apparatus (length, 1 m; width, 2 cm; height, 50 cm). Mice were habituated to a black box (finish point) for 2 min and trained to walk on the beam, after which they were placed at one end of the beam and the time to reach the black box at the other end and the number of slips were recorded.

### Statistical analysis

All statistical analyses were performed using GraphPad Prism version 7.04, SPSS and ImageJ software. All data are presented as means ± SEM. The significance of differences among means was assessed using a one-way analysis of variance (ANOVA) with Dunnett’s post-hoc test or two-way ANOVA with Tukey’s post-hoc test. p-values < 0.05 were considered significant; individual p-values (*p < 0.05, **p < 0.01, ***p < 0.001 and ****p < 0.0001) are shown in figure legends.

## Results

### CSB inhibits α-synuclein fibrillation and cell-to-cell propagation

**Fig. 1:**
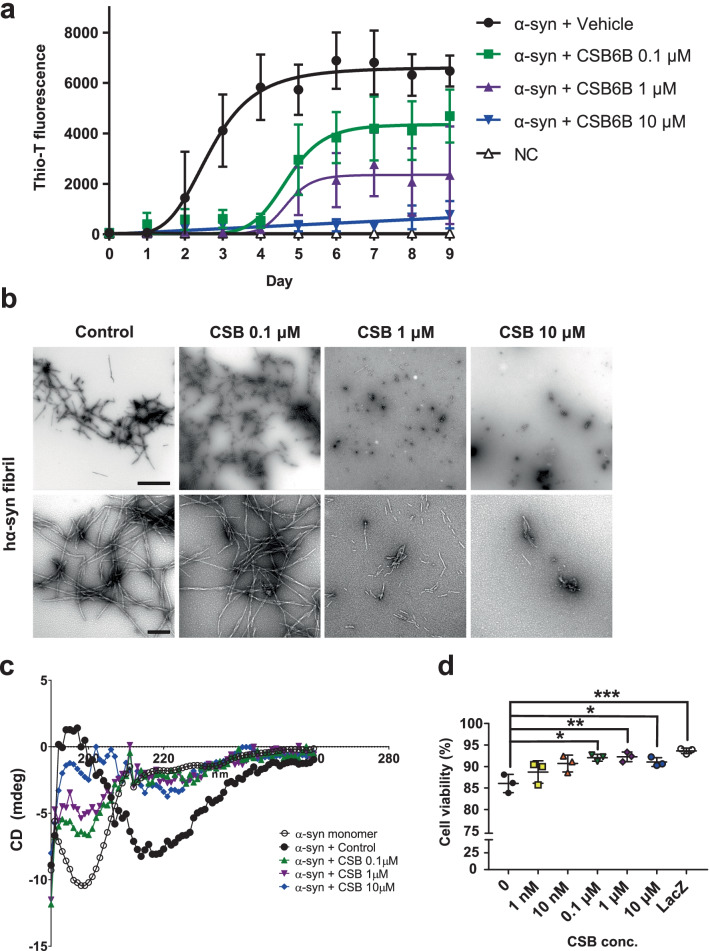
CSB inhibits α-synuclein fibrillation and reduces its toxicity. **a** CSB inhibits α-synuclein fibrillation in a concentration-dependent manner. CSB was incubated with recombinant human α-synuclein for 9 days, and Thio-T fluorescence assays were performed daily. **b** TEM analysis of α-synuclein fibrils incubated with different concentrations of CSB or vehicle (DMSO) control. Scale bars: 1 μm (low magnification, top line) and 200 nm (high magnification, bottom line). **c** Circular dichroism spectra of α-synuclein protein incubated with CSB (0.1 μM, 1 μM and 10 μM) or DMSO (control). **d** CSB (0.1, 1 and 10 μM) significantly improved the viability of α-synuclein–overexpressing cells. Data are presented as means ± SEM (*p < 0.05, **p < 0.01, ***p < 0.001; one-way ANOVA with Dunnett’s post hoc test)

To determine whether CSB inhibits α-synuclein fibrillation, we performed thioflavin-T (Thio-T) binding assays in the presence of different concentrations of CSB (Fig. 1a). Amyloid fibril formation is a typical nucleation-dependent process that shows a sigmoidal relationship in Thio-T binding assays, measured as Thio-T fluorescence as a function of time [21, 22]. Consistent with this, Thio-T fluorescence in the presence of α-synuclein showed a sigmoidal relationship over time indicative of fibrillation (Fig. 1a). CSB inhibited α-synuclein fibrillation in a concentration-dependent manner (Fig. 1a), extending the lag phase of α-synuclein fibrillation and reducing the magnitude of the saturation phase compared with vehicle control (DMSO). The inhibitory effects of CSB were confirmed by electron microscopy and circular dichroism. With increasing amounts of CSB, α-synuclein fibrils became scarcer and shorter (Fig. 1b), and the β-sheet content was reduced (Fig. 1c). When overexpressed in differentiated SH-SY5Y cells, α-synuclein exerted neurotoxicity, reducing cell viability by 25% (Fig. 1d). This cytotoxic effect of α-synuclein was reduced in cells treated with CSB (Fig. 1d).

**Fig. 2:**
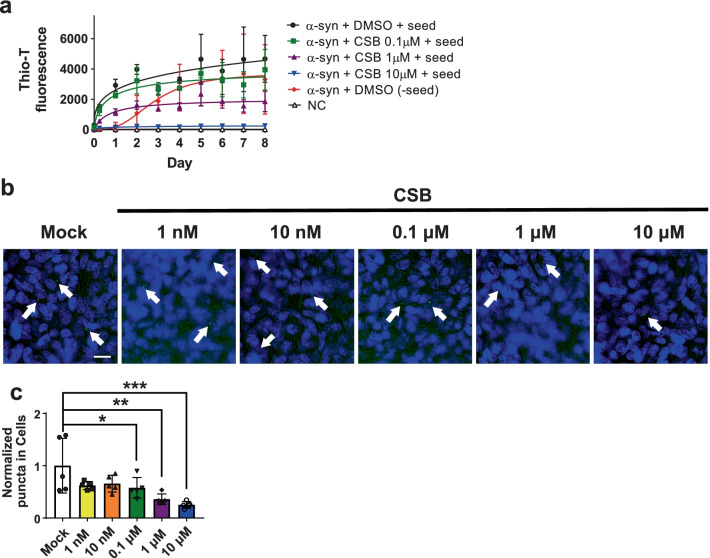
CSB inhibits seeded fibrillation of α-synuclein and reduces its cell-to-cell propagation. **a** Inhibition of α-synuclein fibrillation by CSB in the presence of pre-formed fibril seeds (5%, w/w). **b**, **c** Reduction of α-synuclein aggregate propagation by CSB in the dual-cell BiFC model. Propagated α-synuclein is indicated by white arrows. Scale bar: 20 μm. Data are presented as means ± SEM (*p < 0.05, **p < 0.01, ***p < 0.001)

To assess the effects of CSB on seeded polymerization of α-synuclein, we added α-synuclein seeds containing 5% (w/w) pre-formed fibrils (PFFs) to the fibrillation reaction. The addition of PFFs drastically reduced the lag phase of fibrillation (Fig. 2a). Further addition of CSB to the seeded polymerization reduced the amount of fibril products in a concentration-dependent manner (Fig. 2a). Next, we determined the effects of CSB on cell-to-cell propagation of α-synuclein using the dual cell BiFC cell model [18], in which separate halves of the α-synuclein-conjugated split Venus fluorescent protein are stably expressed in two different cell lines, V1S and SV2. In this model system, cell-to-cell propagation results in both portions of the split system occupying the same cell and reconstitution of Venus fluorescence, which is assessed by monitoring intracellular fluorescent puncta. Addition of CSB inhibited cell-to-cell propagation at concentrations of 0.1, 1 and 10 μM, as evidence by a significant reduction in the formation of fluorescent puncta (Fig. 2b, c). Collectively, these results show that CSB inhibits α-synuclein fibrillation and cell-to-cell propagation.

### CSB binds to the N-terminal region of α-synuclein

**Fig. 3:**
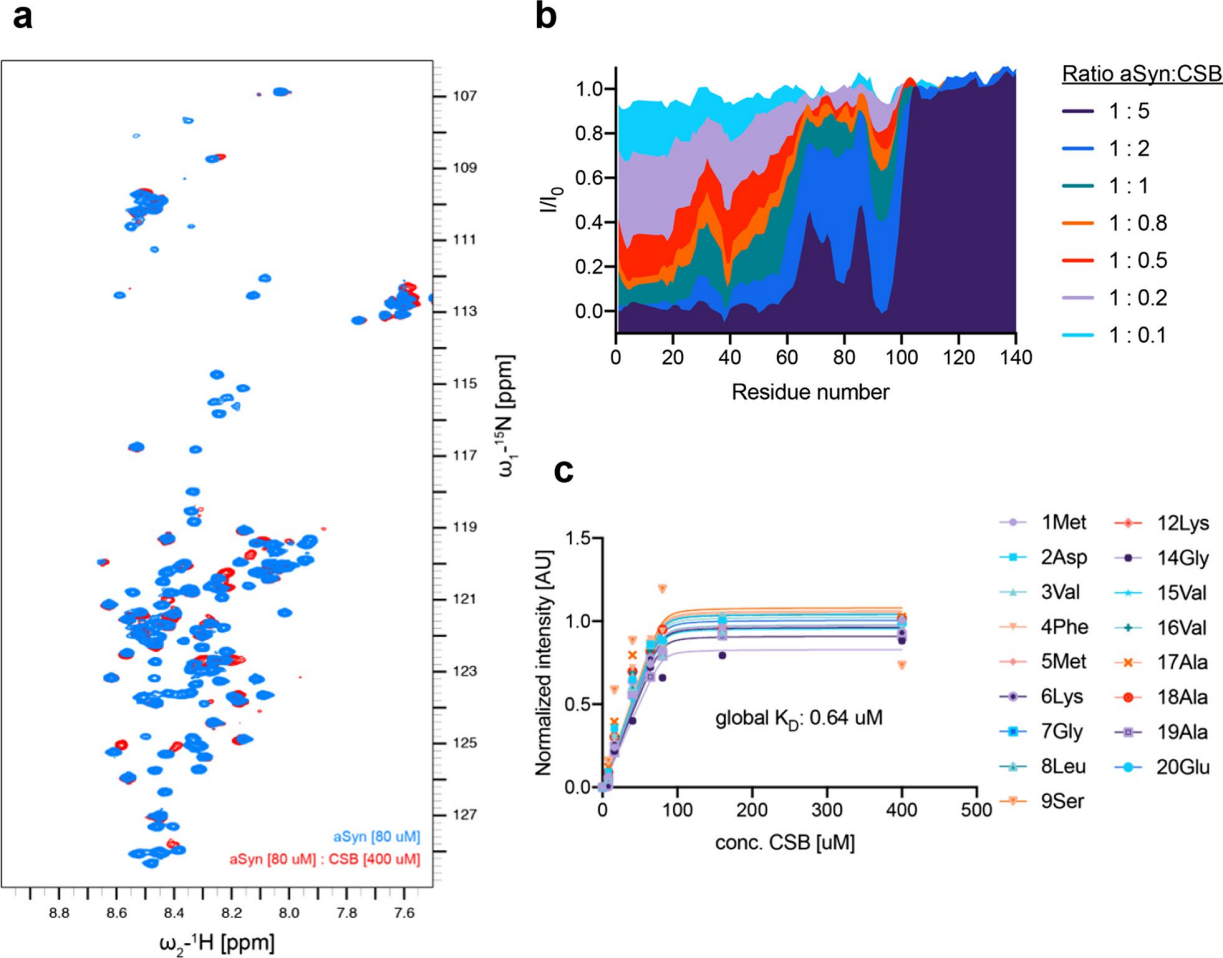
CSB directly binds N-terminal and NAC regions of α-synuclein. **a** Superposition of ^1^H-^15^N HSQC spectra of α-synuclein (80 μM) alone (blue) and in the presence of a fivefold molar excess of CSB (red). Strong signal attenuation together with chemical shift changes were observed for select residues. **b** Residue-specific changes in the intensities of ^1^H-^15^N HSQC cross-peaks of α-synuclein induced at increasing concentrations of CSB. I_0_ and I are the intensities of ^1^H-^15^N HSQC cross-peaks in the absence and presence of CSB, respectively. **c** Intensity changes (I–I_0_) of non-overlapping residues at the N-terminus of α-synuclein reflecting exchange behavior show prominent slowing on the NMR chemical shift scale in the presence of increasing concentrations of CSB. Lines represent global fits to the experimental data for all selected residues based on a reversible 1-to-1 binding model

To determine whether CSB modulates α-synuclein aggregation by directly binding to it, we added increasing concentrations of CSB to ^15^N-labeled α-synuclein monomers. Two-dimensional ^1^H-^15^N correlation spectra of α-synuclein revealed chemical shift perturbations in several signals at substoichiometric CSB concentrations (Additional file 1: Fig. S1). In addition, numerous α-synuclein cross-peaks were strongly broadened at an equimolar CSB concentration (Fig. 3). This broadening was further enhanced at 2- and 5-fold molar excesses of CSB, resulting in the complete loss of signals up to approximately residue 60 (Fig. 3a, b). A global fit of the concentration-dependent decrease in the intensity of non-overlapping cross-peaks originating from residues 1–20 yielded a dissociation constant (K_D_) of approximately 640 nM for the interaction of CSB with α-synuclein (Fig. 3c).

A 5-fold excess of CSB also caused a strong decrease in ^1^H-^15^N signals of residues ~ 60–100, without affecting the acidic C-terminal 40 residues (Fig. 3b). The CSB-induced decrease in signal in the 60–100 region was most pronounced in proximity to phenylalanine 94. In addition, a clear concentration-dependent decrease in intensity was observed for tyrosine 39 and its neighboring residues (Fig. 3b), suggesting that CSB stacks with the rings of aromatic residues in α-synuclein. Taken together, these NMR spectroscopy findings demonstrate that CSB preferentially binds to the N-terminus of α-synuclein, including the hydrophobic NAC (non-amyloid-β component) region that is essential for the pathogenic aggregation of α-synuclein [23, 24].

### CSB alleviates motor behavioral deficits and neuropathology in a mouse model of synucleinopathy

**Fig. 4:**
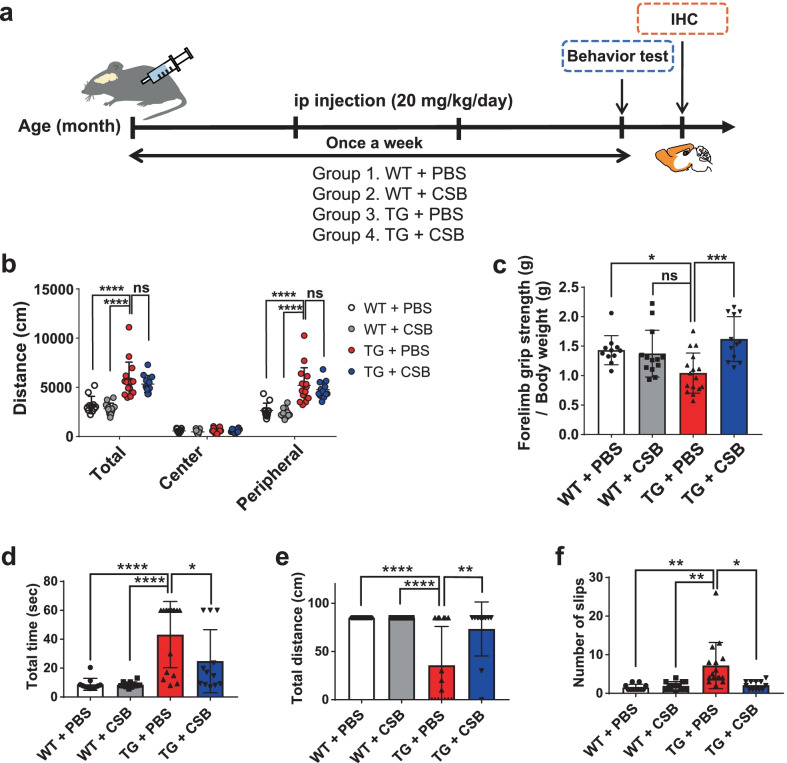
CSB improves motor functions in a transgenic mouse model of synucleinopathy. **a** Schematic representation of the experimental timeline. Six-month-old A53T Tg and WT littermates were intraperitoneally injected weekly with CSB or PBS for 3 months. **b** Open field test. **c** Forelimb grip strength test. **d**–**f** Balance beam test. Data are presented as means ± SEM (*p < 0.05, **p < 0.01, ****p < 0.0001; ns, non-significant difference)

To verify the effects of CSB in vivo, we injected CSB into a mouse model of synucleinopathy and performed motor behavioral tests and neuropathological analyses. For behavioral tests, we performed open field tests, balance beam tests, and forelimb grip strength tests. CSB (20 mg/kg/d) or vehicle (PBS) was intraperitoneally injected into 6-month-old A53T α-synuclein transgenic (Tg) mice and wild-type (WT) littermates for 3 months (Fig. 4a). Body weight, measured weekly, did not differ between Tg mice and littermates, with or without CSB treatment (Additional file 2: Fig. S2a, b). Open field tests, performed to examine locomotive behavior, showed increased locomotion in Tg mice compared with WT littermates (Fig. 4b), consistent with a previous report that these Tg mice exhibit a hyperactive phenotype by 9 months [25]. CSB administration had no effect on this hyperactivity behavior (Fig. 4b). A subsequent examination of forelimb grip strength showed significantly reduced forelimb grip strength in Tg mice, a phenotype that was rescued by CSB treatment a (Fig. 4c). To test motor coordination, we then performed balance beam tests, measuring total time spent, total distance, and number of slips. As shown in Fig. 4d–f, Tg mice showed reduced motor coordination in all parameters tested. Again, CSB administration rescued these motor coordination deficits in Tg mice.

**Fig. 5:**
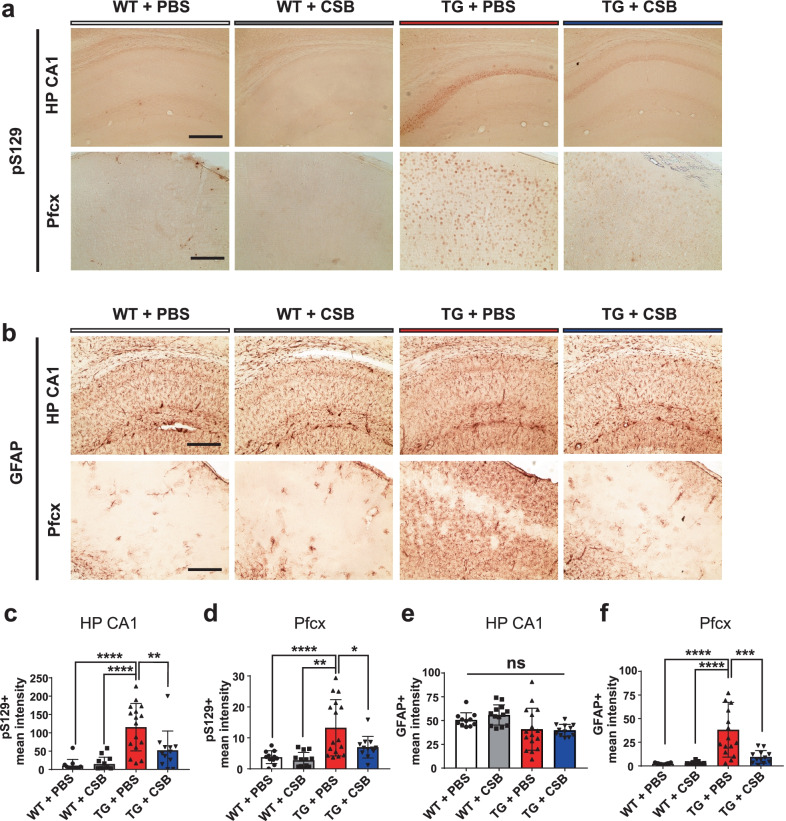
CSB reduces S129-phosporylated α-synuclein levels and astrogliosis in a transgenic mouse model of synucleinopathy. **a**, **b** Representative images showing immunohistochemical staining for S129-phosphoryated α-synuclein (pS129) (**a**) and GFAP (**b**). HP, hippocampus; Pfcx, prefrontal cortex. **c**, **d** Quantification of pS129 levels in the HP (**c**) and Pfcx (**d**). **e**, **f** Quantification of GFAP levels in the HP (**e**) and Pfcx (**f**). Scale bar: 50 μm. Data are presented as means ± SEM (*p < 0.05, **p < 0.01, ***p < 0.001, ****p < 0.0001; ns, non-significant difference.)

Finally, we assessed neuropathological lesions in these Tg mice by examining the deposition of S129-phosphorylated α-synuclein (pS129) and astrogliosis (Fig. 5). This Tg model has been shown to exhibit increased pS129 in several brain regions, including the hippocampus and motor cortex, and higher levels of astrogliosis with increased glial fibrillary acidic protein (GFAP) immunoreactivity [26]. Consistent with this, we also found elevated pS129 levels (Fig. 5a) and GFAP immunoreactivity in the hippocampus and prefrontal cortex of Tg mice (Fig. 5b). CSB administration significantly reduced pS129 levels in both regions and GFAP immunoreactivity in the prefrontal cortex (Fig. 5c, d); it also tended to decrease GFAP immunoreactivity in the hippocampus, although this difference failed to reach statistical significance (Fig. 5e). Collectively, these in vivo data suggest that CSB administration alleviates the behavioral deficits and neuropathological lesions caused by α-synuclein expression in the brain.

## Discussion

In the present study, we demonstrated that CSB inhibits both de novo and seeded aggregation of α-synuclein. CSB directly binds to the amphipathic N-terminal (1–60) and, to a lesser extent, the NAC (61–95) regions of α-synuclein. In a cell model, CSB inhibited cell-to-cell propagation of α-synuclein, and administration of CSB to a transgenic model of synucleinopathy resulted in alleviation of behavioral deficits and neuropathological features. These findings suggest that small molecules, such as CSB, that bind the N-terminal region of α-synuclein could have therapeutic potential for PD and related synucleinopathies.

In previous studies, dyes, such as phthalocyanine tetrasulfonate (PcTS), Congo red and Coomassie brilliant blue R (CBBR), that bind the N-terminal parts of α-synuclein have been shown to inhibit α-synuclein fibrillation [27–29]. Congo red exerts its inhibitory action through binding to N-terminal and NAC regions of α-synuclein [30]. In addition, PcTS, which was previously shown to exhibit anti-scrapie function, inhibits α-synuclein fibrillation by binding to specific sites in the N-terminus of α-synuclein [31, 32]. Similar to these previously identified fibrillation inhibitors, CSB binds with high affinity to the N-terminus of α-synuclein and with lower affinity to the NAC region. The amphipathic N-terminal region of α-synuclein has a highly conserved lysine-rich repeat motif and interacts with lipid membranes. It is not clear how the interaction between CSB and the N-terminus of α-synuclein inhibits the fibrillation process. One possibility is that this interaction sterically interferes with protein–protein interactions between α-synuclein molecules mediated by fibril core regions. Using solid-state NMR, previous studies showed that the β-sheet-rich α-synuclein fibril core comprises residues 38–98 [33, 34]. This fibril core overlaps with the CSB binding site, identified in this study using NMR spectroscopy. The effectiveness of small molecules with high affinity for the N-terminus suggests the hypothesis that the initial focal points for fibrillation reside in this region. Another potential mechanism of action for CSB in the cellular milieu is related to its effects on membrane binding. Several studies have shown that modifications of the N-terminal region of α-synuclein critically affect aggregation and membrane binding [24, 35, 36]. Mutations in a 7-residue (36–42) region in the N-terminus of α-synuclein modify age-dependent α-synuclein aggregation by altering lipid binding properties in a *Caenorhabditis elegans* model [24]. Collectively, these observations suggest that N-terminal–binding molecules inhibit α-synuclein fibrillation by interfering with self-assembly of the protein and/or altering its interaction with lipid membranes.

Interestingly, CSB is a competitive inhibitor of Aβ binding to PrP^c^, the cellular form of the prion protein, PrP^sc^ [37]. In addition, it has been shown that intracerebroventricular injection of CSB prevents Aβ-induced neurotoxicity and neuroinflammation by inhibiting NF-κB and NLRP3 in AD model mice [38]. CSB also exhibits modulatory effects on various brain activities and neurological behaviors. For example, CSB inhibits neurotransmitter uptake into synaptic vesicles and modulates ATP dependent-vesicular membrane potential in the rat brain [39]. It also alleviates acute and chronic hyperactivity behavior induced by subcutaneous injection of methamphetamine [40]. Therefore, CSB appears to have multi-functional effects on neurological conditions beyond its anti-aggregation properties.

CSB itself has limitations as a central nervous system-targeting drug because it is highly water-soluble and may not have a good bioavailability. Therefore, if CSB is to be developed as a neurological drug, it will need to be modified to improve its delivery. In this context, CSB-coated liposomes have demonstrated improved delivery to target organs and diminished systemic side effects in high-dose therapy [41]. Further studies are warranted to achieve effective delivery of CSB and its derivatives to the brain.

In conclusion, our study demonstrated that CSB inhibits fibrillation of α-synuclein through interaction with the N-terminus of the protein and thereby inhibits cell-to-cell propagation of the protein. Modifications of CSB to improve its bioavailability and brain delivery might be a promising approach for development of a new drug for PD and related synucleinopathies.

## Supplementary Information

**Additional file 1: Figure S1. **Addition of CSB to α-synuclein induces changes in the positions of cross-peaks of selected residues in ^1^H-^15^ N HSQC spectra of α-synuclein. Addition of CSB to α-synuclein induces chemical shift perturbations in the N-terminus and some peaks in the 60–100 amino acid region of α-synuclein at α-synuclein: CSB ratios > 1:1, reflecting chemical shift perturbations.

**Additional file 2: Figure S2. **Effects of CSB treatment on body weights of mice. (a) Body weights of mice in each group during the 3-month treatment period. Data are presented as means ± SEM. The numbers of mice in experimental groups were as follows: WT + PBS, n = 11; WT + CSB, n = 13; Tg + PBS, n = 16; Tg + CSB, n = 12. (b) Body weight changes in male and female mice.

## Data Availability

All data generated or analyzed during this study are included in this article.
