# Supplementary figures and images for: Chicago sky blue 6B inhibits α-synuclein aggregation and propagation
Source: Mol Brain. 2022 Mar 28;15:27. doi: 10.1186/s13041-022-00913-y (PMC8962151; doi:10.1186/s13041-022-00913-y)

Supplementary Figure 1.

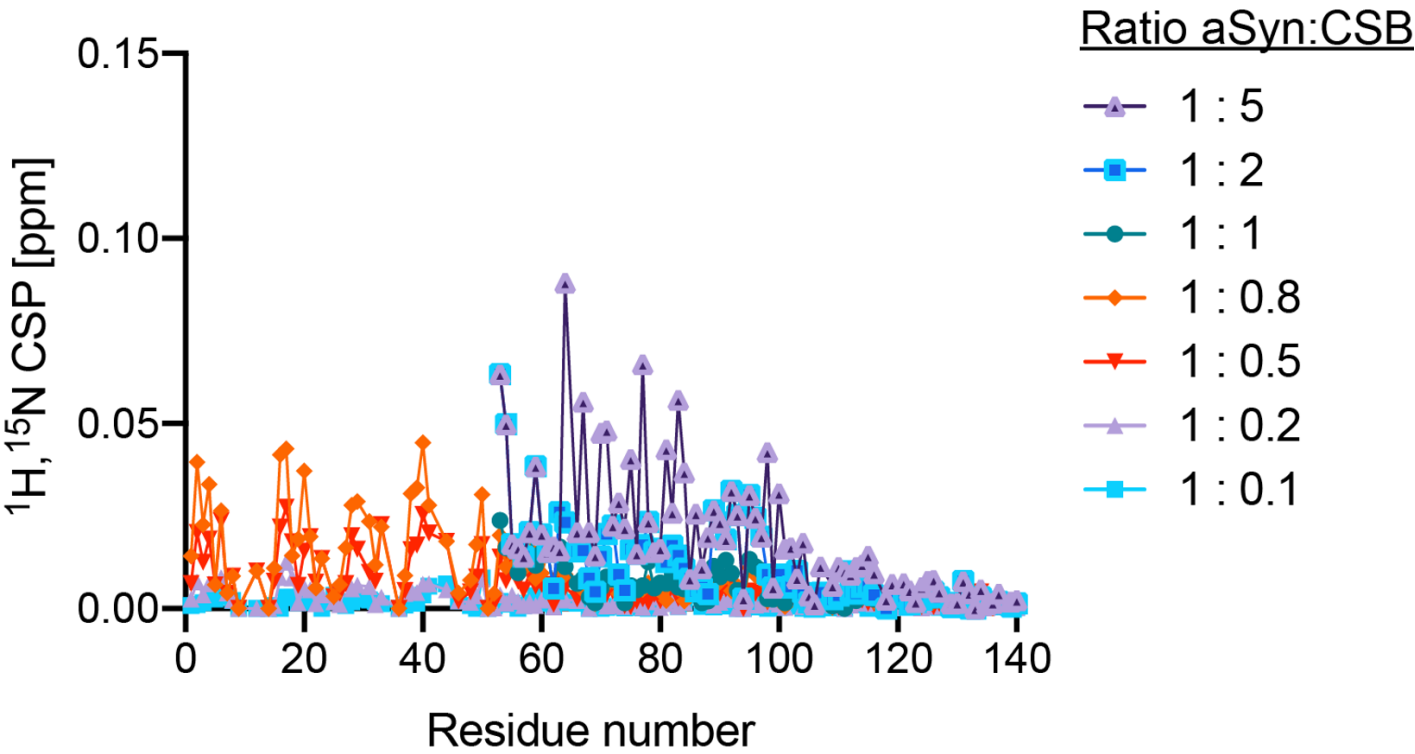

Supplement: Supplementary file 1 — Additional file 1: Figure S1. Addition of CSB to α-synuclein induces changes in the positions of cross-peaks of selected residues in 1H-15 N HSQC spectra of α-synuclein. Addition of CSB to α-synuclein induces chemical shift perturbations in the N-terminus and some peaks in the 60–100 amino acid region of α-synuclein at α-synuclein: CSB ratios > 1:1, reflecting chemical shift perturbations. [file 13041_2022_913_MOESM1_ESM.pdf]

# Supplementary Figure 2.

a

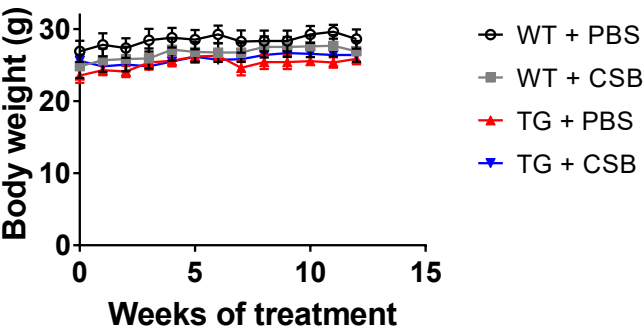

b

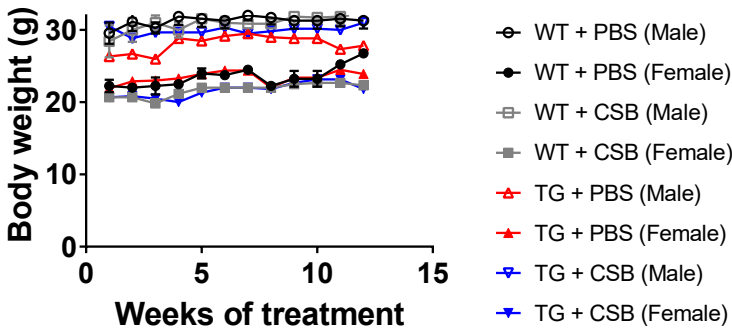

Supplement: Supplementary file 2 — Additional file 2: Figure S2. Effects of CSB treatment on body weights of mice. (a) Body weights of mice in each group during the 3-month treatment period. Data are presented as means ± SEM. The numbers of mice in experimental groups were as follows: WT + PBS, n = 11; WT + CSB, n = 13; Tg + PBS, n = 16; Tg + CSB, n = 12. (b) Body weight changes in male and female mice. [file 13041_2022_913_MOESM2_ESM.pdf]
